# The Role of Oral Ascorbic Acid Administration in Combination With IV N-acetylcysteine in Delaying Inflammatory Cascade in Sepsis: A Case Report

**DOI:** 10.7759/cureus.49868

**Published:** 2023-12-03

**Authors:** Mahmoud Abdo, Neveen Kohaf, Mohamed A Hammad, Chong C Ping

**Affiliations:** 1 Clinical Pharmacy, School of Pharmaceutical Sciences, Universiti Sains Malaysia, Georgetown, MYS; 2 Clinical Pharmacy, Al-Azhar University, Cairo, EGY; 3 Clinical Pharmacy, Al Baha University, Al Baha, SAU

**Keywords:** n-acetylcysteine, pneumonia, sepsis, inflammatory cascade, ascorbic acid

## Abstract

Sepsis is a life-threatening emergency that arises owing to a dysregulated host response to infection, leading to existence organ dysfunction. Vitamin C administration has led to a lower mortality rate in sepsis. N-acetylcysteine (NAC) treatment during sepsis improves hepatic function and enhances tissue oxygenation. The objective of this case report is to investigate the synergistic effect of the combination of vitamin C, thiamine, and NAC in delaying sepsis cascade and prolongation of survival time. In this case report, an oral dose of vitamin C 500 mg three times daily in combination with IV thiamine 100 mg three times daily, IV NAC, and hydrocortisone stress dose resulted in 12 days of survival of an immunocompromised patient with ventilator-associated pneumonia on single anti-pseudomonas beta-lactam antibiotic. The patient was a 60-year-old Malay female with previous bone marrow transplantation surgery and a medical history of ischemic stroke on phenytoin and valproate therapy. The patient was transferred to a medical ward in Penang General Hospital, Malaysia, due to community-acquired pneumonia. She was on ceftriaxone for five days, then sedated and ventilated in the ICU, with a shift to cefepime for three days, which was then changed to meropenem for nine days until the last day of life. Total anti-pseudomonas coverage was 12 days. The patient had multiple comorbidities from phenytoin-induced hepatic encephalopathy, acute kidney injury, and three sessions of hemodialysis. IV vitamin C was not available, so an oral dose was administered with potential efficacy in delaying the sepsis inflammatory cascade, leading to the use of a single (not double) anti-pseudomonas antibiotic for 12 days. Prolonged survival duration may be expected in the case of normal bone marrow patients with ventilator-associated pneumonia sepsis. In conclusion, Vitamin C, thiamine, and NAC combination resulted in delayed sepsis progression for 12 days and the survival of the immunocompromised patient on a single anti-pseudomonas beta-lactam antibiotic.

## Introduction

Sepsis is a life-threatening organ dysfunction caused by a dysregulated host response to infection [1]. It arises owing to a downregulation of reaction to infection, leading to existence system malfunction. The occurrence of sepsis keeps rising in critical care units and hospitals globally: around 31 million sepsis episodes appear globally per year, with nearly 6 million losses. Despite massive breakthroughs in intensive care aid approaches, 30-45 % of cases die upon hospitalization because of septic shock and severe sepsis [2]. Acute respiratory distress syndrome (ARDS) is considered the most critical outcome of sepsis, which is a serious condition of sepsis-induced lung impairment [3]. Sepsis-induced pulmonary injury is correlated with prolonged mechanical ventilation duration and a higher rate of mortality when compared to other types of acute pulmonary injury. Sepsis adversely impacts geriatrics with comorbid conditions, such as impaired immune system and reduced physical state. Pneumonia is the most widespread source of infection for sepsis, accompanied by vascular and intra-abdominal disorders [4].

The expression of inflammatory cytokine and chemokine occurs in sepsis due to the release of reactive nitrogen and oxygen species, as a result of oxidant-induced transcription factors' activation. Oxidants released during sepsis stimulate endothelial malfunction, destroying glycocalyx proteins' surface (e.g., syndecan-1) along with causing cell membrane destruction and enhancing intercellular adhesion molecules' production [5].

Vitamin C is a vitamin that is water soluble and present in all vegetables and fruits. In contrast to many vertebrates, in which l-gulonolactone oxidase catalyzes the last step in the manufacture of vitamin C, humans depend only on a daily diet for the preservation of vitamin C levels in the body [6].

Vitamin C has an antioxidant property, and it is prescribed for methemoglobinemia, burns, scurvy, and healing wounds. Vitamin C participates in oxidation/reduction reactions as well as the metabolic production of catecholamines, steroids, and carnitine. It's also involved in the production of folinic acid from folic acid [7].

There is considerable evidence that an IV vitamin C infusion dose of 50-200mg/kg/24 hours decreased quick sequential organ failure assessment (qSOFA) scores, C-reactive protein (CRP), and procalcitonin (PCT) and contributed to a decreased mortality risk [8]. Thiamine is essential for cellular energy generation and tissue protection from oxidative stress [9]. Recently the combined treatment of vitamin C and thiamine for septic shock has been established [9].

N-acetylcysteine (NAC) is a thiol molecule that possesses antioxidant and vasodilatory characteristics. As a source of sulfhydryl and glutathione groups in cells, NAC is known as a crucial antioxidant. It also acts as a free radical scavenger due to its interaction with reactive oxygen species (ROS). The natural antioxidant glutathione is diminished in septic individuals, which results in lowered protection of cell membranes as compared to oxygen free radicals [10]. NAC functions as a source of glutathione and can replenish intracellular glutathione reserves. Furthermore, NAC affects renal microcirculatory circulation [11]. NAC therapy at the initial hours of septic shock or severe sepsis, which may reduce peroxidative damage, recover tissue oxygenation, and improve hepatic and cardiac functions while delaying administration, had a deleterious impact on critically ill cases with complex organ failure [12].

Synergistic activity of combined vitamin C and NAC has been reported in mutagenesis and carcinogenesis [13] and on human mesenchymal stem cells against mitoptosis, apoptosis, and necroptosis [14]. Herein we see the effect of vitamin C, thiamine, and NAC combination for the first time on sepsis cascade.

## Case presentation

A 60-year-old Malay woman was admitted in April 2018 to the ICU with the chief of complaint postsurgical community-acquired pneumonia. The patient had previous stem cell transplant surgery. The patient had a history of present illness focal seizure secondary to stroke, fever, weakness, hematuria, cholestasis jaundice, acute liver failure secondary to sepsis (coagulopathy, hyperbilirubinemia, and hypoglycemia), acute kidney injury, metabolic acidosis, phenytoin drug reaction with eosinophilia, and systemic syndrome.

The patient was re-admitted after two weeks from stem cell transplant surgery discharge due to community-acquired pneumonia with a medication history of phenytoin, valproate, simvastatin, and aspirin. In ICU on the first day (April 5, 2018), the patient was sedated and ventilated with targeting mean arterial pressure (MAP) above 65 mmHg. Phenytoin and valproate were stopped due to hyperbilirubinemia. Vitamin K was started to correct coagulopathy. Ceftriaxone 2 gm (third generation cephalosporin) once daily was started for community-acquired pneumonia in combination with metronidazole IV for potential risk of aspiration due to past seizures. On the second ICU day (April 6, 2018), the temperature decreased from 38 to 37 Celsius; lactulose was added to exclude potential phenytoin-induced hepatic encephalopathy.

On the third day (April 7, 2018), oral vitamin C was started with a dose of 500 mg three times daily in combination with thiamine 100 mg to prevent oxalate kidney stones until the last day of the patient’s life. Upon day six in the ICU (April 10, 2018), the fever resumed above 38 Celsius and blood pressure decreased to 103/70 (a sign of sepsis). Hence cefepime (fourth-generation cephalosporine) was started instead of ceftriaxone as a single anti-pseudomonal beta-lactam antibiotic for suspected ventilator-associated pneumonia (VAP). On day seven (April 11, 2018), hydrocortisone was started with a stress dose as an anti-inflammatory. On day nine (April 13, 2018), fever rose to 39 Celsius so cefepime and metronidazole combination was substituted with meropenem as a single-agent anti-pseudomonal carbapenem to cover pseudomonas, gram-negative bacteria, and anaerobes. On day 10 (April 14, 2018), the patient was in need of a vasopressor (nor-adrenaline) to raise MAP above 65 mmHg, and the first hemodialysis session was started due to metabolic acidosis.

On day 11 (April 15, 2018), NAC was added to vitamin C, thiamine, hydrocortisone, and a single anti-pseudomonal beta-lactam (meropenem). On day 13 (April 17, 2018), blood culture was sampled to exclude bloodstream infection upon hemodialysis, and the patient was weaned off vasopressors.

On day 14 (April 18, 2018), the hemodialysis second session was started; the temperature rose to 38.5 Celsius, so vancomycin (anti-methicillin-resistant Staphylococcus aureus (anti-MRSA)) was added empirically with renal dose adjustment according to dialysis. On day 17 (April 21, 2018), the patient died due to disseminated intracellular coagulopathy (DIC) during the third hemodialysis session. Table 1 shows the stabilization of MAP above 65 mm/Hg until the last day of survival. Table 2 shows rising Inflammatory marker (WBC) and delayed platelet drop because of DIC.

**Table 1:** MAP was above 65 mm/Hg until the last day of survival. T: temperature; BP: blood pressure; MAP: mean arterial pressure; HR: heart rate; RR: respiratory rate

Date	April 15	April 16	April 17	April 18	April 19	April 20	April 21
Day	11	12	13	14	15	16	17
T (^o^C)	37	37.5	38	38.5	37.5	37	-
BP (mmHg)	107/60	128/63	154/66	145/57	142/55		-
MAP (70 – 105mmHg)	80	76	75	80	75	70	-
HR (60 – 100 b/min)	120	121	120	120	120	120	-
RR (12 – 18 b/min)	11	12	11	11	11	11	-

**Table 2:** Rising Inflammatory marker (WBC) and delayed platelet drop because of DIC Hgb: hemoglobin; HCT: hematocrit; MCV: mean corpuscular volume; MCH: mean corpuscular hemoglobin; MCHC: mean corpuscular hemoglobin concentration; DIC: disseminated intracellular coagulopathy

	Date	April 15	April 16	April 17	April 18	April 19	April 20	April 21
	Normal range							
WBC	5.2 – 12.4 x 10^3^/uL	17	13.3	19	11.4	14.3	27.4	
Hgb	14 – 18 g/dL	8.3	8.2	8	7	8.9		
HCT	42 – 52 %	23.7	23.8	24	21	30.1		
MCV	80 – 94 fL		82.9	83	82.8			
MCH	27 – 31 pg		28	28	27.7			
MCHC	33 – 37 g/dL		34.5	34	34			
Platelet	130 – 400 x10^3^/uL	191	46	55	66	40	44	
Neutrophil	40 – 74 %	35	12	88	80	69.5	20.7	
Lymphocyte	19 – 48 %	1.6	0.9	1.4	1.8	1.2	1.2	

## Discussion

An immunocompromised patient with stem cell transplant survived on single anti-pseudomonal beta-lactam (cefepime then meropenem), not dual anti-pseudomonal agents with two different mechanisms (such as beta-lactam plus aminoglycoside or beta-lactam plus fluoroquinolone as per Infectious Diseases Society of America (IDSA) 2016 guidelines), to treat VAP for 12 days (April 10, 2018 to April 21, 2018) in combination with antioxidants (oral vitamin C and IV NAC) in addition to stress dose of anti-inflammatory steroid. Inflammatory marker WBC decreased from 17 to 13 upon starting of meropenem (with continuing oral vitamin C) on day nine (April 13, 2018) and nor-adrenaline weaned off on April 17, 2018. All cultures had no bacterial growth, so antibiotics were continued empirically.

The expiry of this patient was multifactorial. Previous bone marrow transplantation had hematological complications such as thrombocytopenia, which might accelerate the progress of DIC. The patient withheld phenytoin because of its hematological adverse effects, purpuric dermatitis and eosinophilia, in addition to potential hepatic encephalopathy, which led to acute kidney injury (AKI). Oral vitamin C was administered at a regular dose in combination with thiamine and a single beta-lactam antibiotic (ceftriaxone then single agent anti-pseudomonas cefepime then escalated to meropenem).

Vitamin C given IV to patients suffering from sepsis and septic shock has been shown in several trials to lower mortality [15,16]. Nonetheless, a recently published study concluded that early administration of vitamin C, hydrocortisone, and thiamine in patients suffering from septic shock had no survival benefits in the duration of hospital stays and mortality [17]. Thus, there is now conflicting evidence supporting these conclusions.

In this case report, the authors noted 12 days of survival for an immunocompromised patient on single anti-pseudomonal beta-lactam (not dual-antipseudomonal) for the management of VAP. Guidelines for the treatment of pneumonia in critical care units that were published in 2016 suggested the usage of a beta-lactam anti-pseudomonas antibiotic (like as piperacillin/tazobactam, carbapenems, cefepime) associated with an anti-pseudomonas fluoroquinolone (high doses ciprofloxacin) or an anti-pseudomonas beta-lactam plus an aminoglycoside or aminoglycoside plus fluoroquinolone when beta-lactam cannot be used [18]. On the other hand, another study, Evaluation of Guideline Recommendations for Dual Antipseudomonal Therapy in Hospitalized Adults with Pneumonia, concluded that dual anti-pseudomonas should be locally validated [19], which supports this case report regarding possible effectiveness of the substitution of the second anti-pseudomonas by combination of oral vitamin C, thiamine, NAC and hydrocortisone specially in a normal patient (without bone marrow transplantation like this case report). 

The etiology of this immunocompromised patient’s resilience on single anti-pseudomonas required a multidisciplinary approach. The authors eliminate all causes for survival except the antioxidant effect of oral vitamin C. The efficacy mechanism of vitamin C in pneumonia is by decreasing necrosis factor signaling (NF-kB), decreasing neutrophil extracellular trap formation, and increasing interferon production. These three pathways delay the cascade of reactive oxygen species (oxidative stress), leading to regeneration of the lung-epithelial barrier, improvement of adrenal defense function, and temporary suppression of ARDS [20].

## Conclusions

After a process of elimination, the most likely cause of delayed sepsis progression (due to pneumonia) for 12 days on a single anti-pseudomonas beta-lactam antibiotic is the antioxidant combination (oral vitamin C plus thiamin IV regular dose plus NAC IV regular dose) in addition to hydrocortisone stress dose. This patient had confounding factors that may lead to death: bone marrow transplantation, phenytoin-induced hepatic encephalopathy, hypotension during the third dialysis session, and previous AKI. In the case of patients with normal bone marrow, survival days on single anti-pseudomonas can be increased. Oral vitamin C has potential efficacy if an IV dose cannot be available. Authors recommend more studies about prolonged survival days using IV vitamin C with a single anti-pseudomonas, or oral dose in combination with dual anti-pseudomonas antibiotics in pneumonia.
